# Editorial: Synthetic metabolism for the third-generation (3G) biorefineries

**DOI:** 10.3389/fbioe.2023.1214729

**Published:** 2023-05-23

**Authors:** Qing Wang, Hai He, Wei Xiong, Peijian Cao, Jian Cheng, Lei Zhao

**Affiliations:** ^1^ Key Laboratory of Engineering Biology for Low-Carbon Manufacturing, Tianjin Institute of Industrial Biotechnology, Chinese Academy of Sciences, Tianjin, China; ^2^ National Center of Technology Innovation for Synthetic Biology, Tianjin, China; ^3^ Max Planck Institute for Terrestrial Microbiology, Marburg, Germany; ^4^ Biosciences Center, National Renewable Energy Laboratory, Golden, CO, United States; ^5^ China Tobacco Gene Research Center, Zhengzhou Tobacco Research Institute of CNTC, Zhengzhou, China

**Keywords:** one-carbon feedstock, carbon metabolism, natural products, carbon fixation, cell factory design

The demand for energy has increased significantly to meet the development of the economy and the increasing population. With the continuous consumption of energy, the shortage of non-renewable resources and the greenhouse effect have aroused worldwide attention. Therefore, the conversion of one-carbon substrates such as formate, methanol, and carbon dioxide (CO_2_) as sustainable feedstocks into high-value-added products is a potential way to address such concerns. Although one-carbon substrates can be converted into organics by natural pathways, these pathways may not be optimal due to the multiple enzymes involved and remarkable amounts of energy consumption. Thus, synthetic biology, as an emerging field, offers a new approach to boost one-carbon bioconversion.

Microorganisms are widely used in one-carbon biotransformation by synthetic biology because of their rapid growth and amenable genetic manipulation. In addition, plants are becoming ideal and sustainable platforms for one-carbon utilization due to their advantages of post-translational modifications and compartmentalization. However, efficient bioconversion from one-carbon substrates to high-value-added products still needs a long-term development process in which a complete understanding of biosynthetic pathways and potential regulatory mechanisms remains to be discovered. In particular, the efficient production of high-value-added products requires high adaptability of metabolic pathways and the chassis.

This Research Topic contains a collection of original research papers and reviews that aim to cover the latest and most novel research trends and methods for improving the bioconversion of one-carbon substrates to high-value-added products in engineered organisms to achieve a globally sustainable future.

Wefelmeier et al. provide a toolbox to optimize gene expression to promote yeast *Ogataea polymorpha* used as a chassis for biological production processes. The authors evaluate the activity of different promoters with methanol, glucose, and glycerol as carbon sources and a combination carbon source by GFP fluorescence signal. Meanwhile, the influence of terminators on gene expression was also assessed by GFP under methanol, glucose, and glycerol carbon sources. Two terminator constructs that showed the highest and the lowest GFP expression were selected to analyze the mRNA abundance and stability to provide preliminary evidence on how terminators affect gene expression in *O. polymorpha*. Finally, two promoters were coupled with three terminators by expressing two different reporter genes to confirm that promoters and terminators could be used as independent genetic elements. Therefore, the toolbox of promoters and terminators for *O. polymorpha* was established and will be extended by characterizing additional elements.

Wang et al. describe a systematic review of progress toward efficiently increasing CO_2_ fixation via the design of photorespiratory bypasses in plant chassis for bioproduction. The authors summarize five photorespiratory bypasses that have been assembled and implemented in plants and evaluate the characteristics of each bypass and their impacts on plants. In this review, some potential bypasses that can be tested in plants are also described, including glycolate decarboxylation, recycling glycolate without CO_2_ release, and carbon-positive photorespiratory shunts. Finally, the authors discuss the future perspective on the modification of photorespiration by synthetic biology. These advances provide important enlightenment for the progressive development of possible strategies for photorespiration improvement to enhance carbon fixation by synthetic biology in plants.

Kim et al. report an optimized *Escherichia coli* strain that achieved lactate production from formate. The authors utilize the previously engineered formatotrophic strain that was obtained by equipping it with the reductive glycine pathway. The adaptive laboratory evolution approach was used to select the faster-growing strain for genome sequencing under culture conditions with formate and CO_2_ as the sole carbon sources. The sequencing results showed that the blocking of acetate metabolism is the reason for the improved growth of the strain. Thus, the engineered strain was obtained by deleting two genes of acetate biosynthesis. The biological transformation of formate to lactate was achieved by deleting the lactate assimilation enzyme and transforming lactate dehydrogenase in this engineered strain. This research achieves the industrial application of the reductive glycine pathway and will fully realize the full potential of the reductive glycine pathway by strain engineering and establishing an optimized biological process.

Aiming at the bottleneck of methionine production that lacks methyl donor supply, Shen et al. propose a novel strategy to construct three exogenous modules for methionine biosynthesis. The authors construct a methanol module and introduce it into an engineered *E. coli* strain that produces methionine. However, although three modifications were introduced to methanol dehydrogenase, the toxicity of methanol still had a significant effect on fermentation. Thus, a formate module was developed and assembled into the engineered strain, which increased methionine production, but the conversion rate was lower. To enhance the methyl donor supply, a betaine module was constructed and exhibited a higher capability for methionine biosynthesis. However, the conversion efficiency of the betaine module introduction was still low. Therefore, the improvement of exogenous enzyme activity and the optimization of exogenous module expression need to be explored to improve the biosynthesis of methionine and contribute to the industrial production of methionine.

Taken together, the articles in this Research Topic covered toolbox development in one-carbon bioconversion, utilization of artificial carbon sequestration pathways, and efficient synthesis of natural products in chassis. One-carbon bioconversion research will continue to address current challenges in synthetic biology and promote its applications in industrial production.

